# FDISCO+: a clearing method for robust fluorescence preservation of cleared samples

**DOI:** 10.1117/1.NPh.8.3.035007

**Published:** 2021-09-09

**Authors:** Peng Wan, Yusha Li, Jingtan Zhu, Jianyi Xu, Xiaomei Liu, Tingting Yu, Dan Zhu

**Affiliations:** a Huazhong University of Science and Technology, Britton Chance Center for Biomedical Photonics, Wuhan National Laboratory for Optoelectronics, Wuhan, China; b Huazhong University of Science and Technology, MoE Key Laboratory for Biomedical Photonics, School of Engineering Sciences, Wuhan, China

**Keywords:** optical clearing, FDISCO+, fluorescence preservation, room temperature, fluorescence imaging, antioxidant

## Abstract

**Significance:** The recently reported solvent-based optical clearing method FDISCO can preserve various fluorescent signals very well. However, the strict low-temperature storage condition of FDISCO is not conducive to long-time or repetitive imaging usually conducted at room temperature. Therefore, it is important to solve the contradiction between fluorescence preservation and imaging condition.

**Aim:** We develop a modified FDISCO clearing method, termed FDISCO+, to change the preservation condition from low temperature to room temperature.

**Approach:** Two alternative antioxidants were screened out to effectively inhibit the peroxide generation in the clearing agent at room temperature, enabling robust fluorescence preservation of cleared samples.

**Results:** FDISCO+ achieves comparable fluorescence preservation with the original FDISCO protocol and allows long-time storage at room temperature, making it easier for researchers to image and preserve the samples.

**Conclusions:** FDISCO+ is expected to be widely used due to its loose operation requirements.

## 1 Introduction

It has always been a goal of biologists to obtain three-dimensional (3D) structures of large tissues and organs at high resolution, especially when it comes to reconstructing complex networks, like neural and vascular systems.^1^^–^^5^ Recently, the technique of “optical sectioning” provides a powerful tool for repetitive and efficient 3D tissue imaging.^6^^–^^8^ However, due to the cloudy features of biological tissue, imaging depth is limited among numerous optical fluorescence microscopes such as the confocal microscope, multi-photon microscope, and light-sheet microscope.^1^^,^^9^^–^^12^ In recent years, this problem has been partially solved thanks to the rapid development of optical clearing methods, which can reduce the light scattering and light absorption in the tissues to achieve deep imaging in combination with optical fluorescence microscopy.^13^^,^^14^

Over the years, a variety of optical clearing methods have been developed.^15^^,^^16^ They are usually divided into three categories: the hydrophobic clearing methods, such as BABB,^17^ 3DISCO,^18^^–^^20^ iDISCO,^21^ uDISCO,^22^ FluoClearBABB,^23^ Ethanol-ECi,^24^ vDISCO,^25^ sDISCO,^26^ a-uDISCO,^27^ PEGASOS,^28^ and FDISCO;^29^ the hydrophilic clearing methods, such as Sca*l*eS,^30^ SeeDB,^31^^,^^32^ *Clear^T^*,^33^ CUBIC,^34^^–^^36^ C_e_3D,^37^ RTF,^38^ and MACS;^5^ and the hydrogel-based clearing methods, such as CLARITY^39^^,^^40^ and PACT-PARS.^41^^–^^43^ Different clearing methods are usually applied for certain scopes, and they have respective strengths and weaknesses: the aqueous-based clearing methods usually show good fluorescence preservation, and the organic solvent-based clearing methods usually have excellent clearing capability.^44^

The solvent-based clearing methods are widely used due to their characteristics of reduced sample size and high transparency, as size reduction can break the limit of working distance of objective to image larger volumes, and high transparency allows imaging deeper.^4^^,^^22^ 3DISCO, as an early published method, has advantages on both short clearing time and high tissue transparency. However, 3DISCO is not compatible with endogenous fluorescent molecules.^19^^,^^22^ To address this issue, FDISCO was recently proposed by modifying the experimental conditions of 3DISCO and realizes the preservation of the endogenous fluorescence signals, expanding the application scope of DISCO methods.^29^ However, FDISCO requires a strict storage condition that the cleared samples must be stored at low temperature (LT) (4°C to 8°C) because the fluorescence signals would be rapidly and severely quenched at room temperature (RT), whereas the imaging apparatus of conventional fluorescence microscopy is generally under RT environment during imaging. As a compromise between the fluorescence preservation and the imaging temperature, the FDISCO-cleared samples must be imaged in a short time. But contradictions are inevitable when it comes to long-time or repetitive imaging operation. In this case, the fluorescence would decrease; hence imaging at RT might influence the imaging quality and the visualization of tissue structures.

In this study, we developed FDISCO+, a clearing method that optimizes the preservation condition of FDISCO from LT to RT. The FDISCO+ achieves comparable fluorescence enhancement with FDISCO and preserves the endogenous fluorescence at RT for a long time, making it easier for researchers to image and store the samples. Due to its loose operation requirements, FDISCO+ is expected to be widely used.

## 2 Methods

### 2.1 Animals

In this study, we used *Thy1*-GFP-M mice, *CX3CR1*-GFP mice, and C57BL/6J mice (6 to 12 weeks old). Mice were anesthetized with a mixture of 2% α-chloralose and 10% urethane (0.8  mL/100  g) via intraperitoneal injection. Next, mice were transcardially perfused with 0.01 M phosphate-buffered saline (PBS) (P3813, Sigma-Aldrich) and then 4% paraformaldehyde (PFA) (158127, Sigma-Aldrich). Finally, the brains were dissected and postfixed overnight at 4°C in 4% PFA. Some brains were sliced into 1-mm-thick or 2-mm-thick coronal sections with a vibratome (Leica VT 1200s). The animal care and experimental protocols were in accordance with the Experimental Animal Management Ordinance of Hubei Province, China and have been approved by the Institutional Animal Ethics Committee of Huazhong University of Science and Technology.

### 2.2 Chemicals and Reagents

In this study, we used the following chemicals: tetrahydrofuran (THF) (186562, Sigma-Aldrich), dibenzyl ether (DBE) (108014, Sigma-Aldrich), $N,N,N^{\prime },N^{\prime }$-tetrakis (2-hydroxypropyl) ethylenediamine (EDTP) (T0781, Tokyo Chemical Industry Co., Ltd.), DL-alpha-tocopherol (vitamin E) (A17039, Alfa Aesar), and triethylamine (80134318, Sinopharm Chemical Reagent Co. Ltd.).

For dehydration solutions, THF was mixed with ${\mathrm{dH}}_{2}O$ according to the following concentration: 50% (v/v), 70% (v/v), 80% (v/v), and 100% (v/v). Triethylamine was added to adjust the pH to about 9.0. For clearing agents, the eFDISCO+ was prepared by adding 0.5% (w/v) EDTP to the DBE, and the vFDISCO+ was prepared by adding 0.5% (v/v) vitamin E to the DBE.

The column absorption chromatography with basic activated aluminum oxide (20001861, Sinopharm Chemical Reagent Co. Ltd.) was used to remove the peroxides produced in THF and DBE, as described in the literature.^19^^,^^20^

### 2.3 Measurement of Peroxide Concentration

The basic activated aluminum oxide was used to remove the peroxides in DBE. Then EDTP at concentrations of 0.1% (w/v), 0.5% (w/v), 1% (w/v), and 2% (w/v); vitamin E at concentrations of 0.1% (v/v), 0.5% (v/v), 1% (v/v), and 2% (v/v); and triethylamine at concentrations of 0.1% (v/v), 0.5% (v/v), 1% (v/v), and 2% (v/v) were added to 10 mL DBE in 50 mL centrifugal tubes (430829, Corning, USA) to prepare different refractive index (RI) matching solutions, respectively. Quantofix-25 strips (Macherey-Nagel, Germany) were used to measure the peroxide concentration of the reagent after 0, 9, 24, and 72 h. All tubes except the pure DBE group at LT were placed horizontally in a dark room at RT. The pure DBE group at LT was placed in a refrigerator at 4°C to 8°C.

### 2.4 FDISCO+ Clearing Procedure

The 1-mm-thick brain sections were dehydrated with THF solutions at graded concentrations: 50% (v/v), 70% (v/v), 80% (v/v), and 100% (v/v) (twice), with 1 h each step at 4°C to 8°C. Then the dehydrated samples were transferred to the RI matching solutions for 30 min at RT.

For whole-brain clearing, the samples were dehydrated with 50% (v/v), 70% (v/v), 80% (v/v), and 100% (v/v) (thrice) THF solutions, with 12 h each step at 4°C to 8°C, followed by immersing in the RI matching agents for more than 3 h at RT.

During the clearing, the samples were covered with aluminum foil to ensure a dark environment, and all steps were performed with slight shaking. The cleared samples were stored at RT all the time.

### 2.5 Fluorescence Labeling

For nuclei staining, the 1-mm-thick brain sections were incubated with 1% (w/v) propidium iodide (PI) (P1304MP, Life Technologies)/PBST (0.2% Triton X-100/PBS) for 24 h at RT with slight shaking. Then sections were washed several times with PBST for 6 h.

For vasculature labeling, 10  μg Alexa Fluor 647 conjugated anti-mouse CD31 antibody (CD31-A647) (102416, BioLegend) was used to label the vasculature in C57BL/6J mouse brain by caudal vein injection. The mouse was perfused 30 min after injection. The postfixed mouse brain was sliced into 1-mm-thick coronal sections.

### 2.6 Measurement of Transparency

A digital camera (HDC-HS900GK) was used to acquire the bright-field images of samples. We used a visible-near-infrared optical fiber spectrometer (USB4000, Ocean Optics, USA) to produce a circular spot of light (diameter, 5 mm) to irradiate the samples immersed in the cleared medium. Then the transmitted light was measured on the other side of the samples. The light across only the clearing medium was measured as the blank value. Finally, the light transmittance was normalized by the blank value.

### 2.7 Fluorescence Imaging

For brain slices, the cortical regions of the samples were imaged with a confocal fluorescence microscope (LSM 710, Zeiss, Germany) equipped with objectives of Fluar 5×/0.25 (dry; working distance, 12.5 mm) and Fluar 10×/0.5 (dry; working distance, 2.0 mm). Before and after clearing, the imaging parameters were kept the same when imaging the same regions.

For whole brains, samples were imaged with a customized Bessel light-sheet fluorescence microscope (LSFM) based on Olympus MVX10 and equipped with an sCMOS camera (Hamamatus Flash 4.0 V2), as described in the published literature.^45^ In this study, 3 h to 4 h are needed for imaging per whole mouse brain.

### 2.8 Data Analysis

The Fiji (Version 1.51n), MATLAB (Version 2014a, MathWorks), and Imaris (Version 7.6, Bitplane AG) were used for image processing and quantitative analysis of data. The analysis was derived from the literature.^44^^,^^46^

For the quantification of GFP fluorescence in *CX3CR1*-GFP mouse brain, the maximum intensity projections (MIPs) of 25  μm thickness were acquired from each z stack. We used Otsu algorithm for image thresholding segmentation and binarization.^47^ Then we used MATLAB to extract the signal value and calculate the mean fluorescence intensity of the MIP images. The normalized fluorescence intensity was calculated by dividing the mean fluorescence intensity after n (n=3, 6, 9) h by the value after 0 h. The processing is similar for quantification of PI signals, CD31-A647 signals, and GFP fluorescence for long-time preservation. And the normalized value of PI and CD31-A647 signals was described as the ratio of the mean fluorescence intensity before and after clearing, and the normalized GFP fluorescence intensity for long-time preservation was calculated by dividing the mean fluorescence intensity after n (n=3, 7, 14) days by the mean fluorescence intensity after 0 day.

For the quantification of GFP fluorescence enhancement in *Thy1*-GFP-M mouse brain, we used Fiji to outline the same neurons in the cortical areas before and after clearing and measure the mean fluorescence intensity. The selective neurons must be located in the shallow position to avoid the influence of light scattering on the fluorescence intensity. The normalized mean fluorescence intensity was described by dividing the intensity of cleared group by the value of the uncleared group.

For the LSFM images, we used Fiji to transform the data to 8-bit images and then stitched the images using the Fiji plugins of Grid/Collection Stitching. Finally, we used Imaris to visualize the 3D-rendered images.

## 3 Results

### 3.1 Development of FDISCO+ by Inhibiting the Generation of Peroxides in DBE

**Fig. 1:**
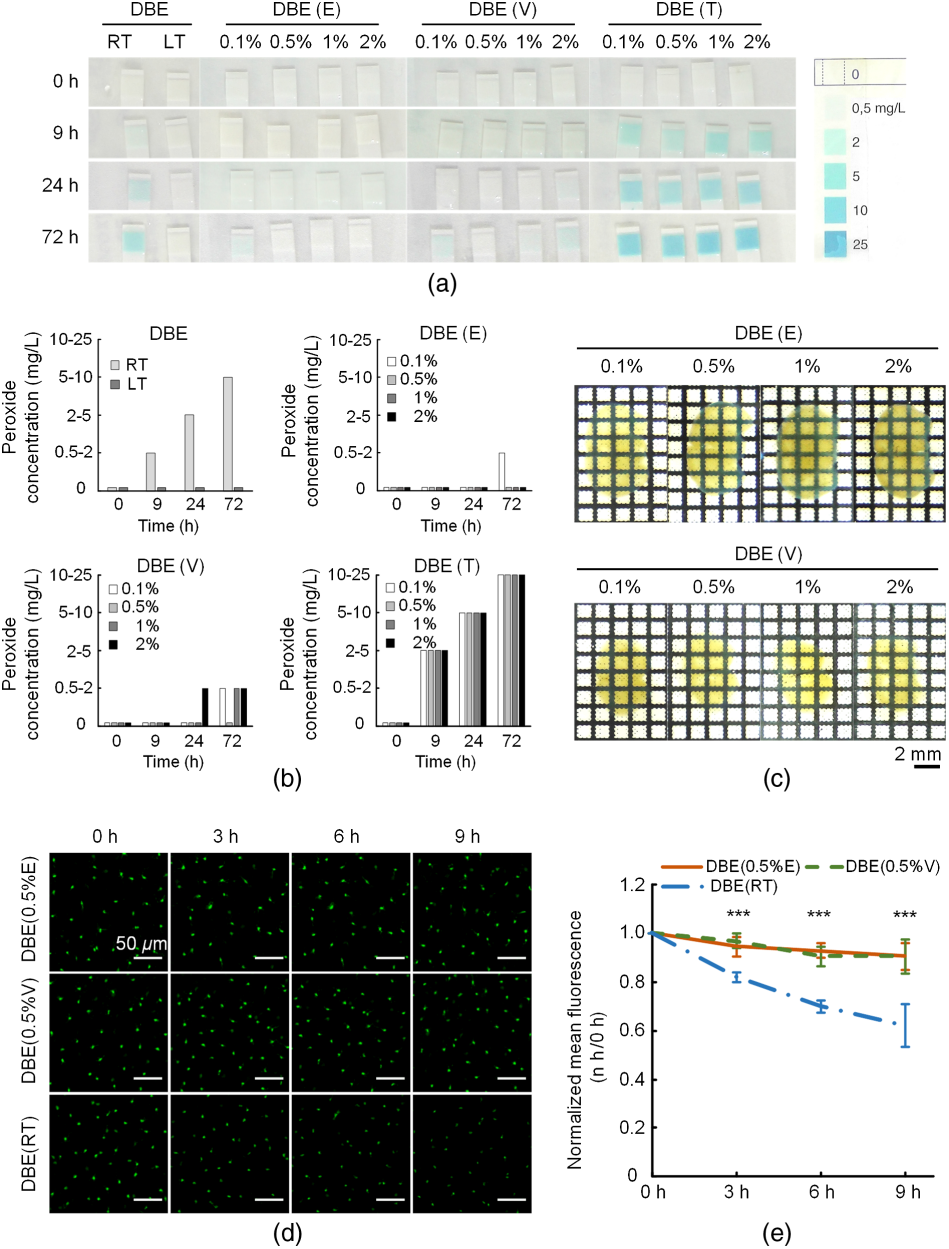
Inhibiting the peroxide generation in DBE at RT preserves the GFP fluorescence. (a) The concentration of peroxides in DBE in different experimental conditions at 0, 9, 24, and 72 h, respectively. RT: the pure DBE was stored at RT; LT: the pure DBE was stored at LT; DBE (E): the DBE was added with EDTP and stored at RT; DBE (V): the DBE was added with vitamin E and stored at RT; and DBE (T): the DBE was added with triethylamine and stored at RT. (b) The quantification of peroxide concentration in (a). (c) 2-mm-thick C57BL/6J mouse brains were cleared in DBE with different concentrations of EDTP or vitamin E. (d) The cleared adult *CX3CR1*-GFP mouse brain slices were put in RI matching solutions at RT and were imaged at 0, 3, 6, and 9 h with the confocal microscope. (e) The decay curves of fluorescence intensity in (d) (n=8). All values are presented as the mean ± SD. Statistical significance in (e) (***p<0.001) was assessed by one-way ANOVA followed by the Bonferroni *post hoc* test.

The peroxide contaminations generated in DBE would quench the endogenous fluorescence severely even at a low concentration.^19^ FDISCO realizes the good preservation of fluorescence in DBE by controlling the temperatures at a low level.^29^ In this work, we measured the peroxide concentration in DBE using Quantofix-25 strips. As shown in Figs. 1(a) and 1(b), the DBE at RT produced about 0.5  mg/L peroxides in 9 h, whereas the DBE at LT did not produce peroxides in 72 h. This result indicates that LT can effectively reduce the generation of peroxides in DBE, facilitating fluorescence preservation. Hence, the samples cleared by FDISCO have to be kept at LT all the time to minimize fluorescence loss of fluorescence intensity for follow-up imaging, especially for the samples that needed repetitive imaging. However, it is not easy to achieve LT during imaging, as the imaging apparatus is usually under RT environment.

To solve the conflict between fluorescence preservation and imaging temperature, we look for a modified protocol based on FDISCO to preserve endogenous fluorescence well at RT. First, we chose three reagents, including EDTP, vitamin E, and triethylamine and tested their antiperoxidation capability when added to DBE. As shown in Figs. 1(a) and 1(b), DBE added with EDTP at concentrations higher than 0.5% (w/v) did not produce peroxides for 72 h, indicating effective inhibition of the peroxide generation in DBE at RT. Additionally, for vitamin E, the concentration of 0.5% (v/v) in DBE also did not produce observable peroxides for 72 h. In contrast, DBE added with triethylamine at concentrations of 0.1% (v/v), 0.5% (v/v), 1% (v/v), and 2% (v/v) induced a faster generation of peroxides than the pure DBE group at RT, indicating that triethylamine could not prevent but accelerate the peroxide generation. These results indicate that the addition of proper concentration of EDTP and vitamin E has antiperoxidation capability on DBE. Further, we validated the influence of the addition of different concentrations of EDTP and vitamin E in DBE on tissue transparency. As shown in Fig. 1(c), the tissue transparency reduced slightly as the concentration of EDTP increased but was not affected by vitamin E with different concentrations. Considering both the oxidation resistance and the clearing transparency, 0.5% (w/v) of EDTP or 0.5% (v/v) of vitamin E was chosen as the final optimal concentration.

Then we used the cleared mouse brain slices (*CX3CR1*-GFP) to verify the fluorescence preserving capability of the DBE added with 0.5% (w/v) EDTP or 0.5% (v/v) vitamin E. The cleared samples were immersed in respective clearing agents at RT and imaged every 3 h. For the samples immersed in pure DBE, the fluorescence signal declined to 62% after 9 h. Although for the samples immersed in the DBE added with EDTP or vitamin E, the GFP signals were significant higher than in pure DBE and showed no noticeable decrease along with time due to inhibited peroxide generation, as shown in Figs. 1(d) and 1(e).

Based on the above results, we proposed an optimized clearing protocol for better fluorescence preservation under RT, termed FDISCO+. We devised two schemes: one using DBE containing 0.5% (w/v) EDTP as the clearing medium termed eFDISCO+, and the other using DBE containing 0.5% (v/v) vitamin E termed vFDISCO+.

### 3.2 FDISCO+ Achieves Comparable Fluorescence Enhancement with FDISCO

**Fig. 2:**
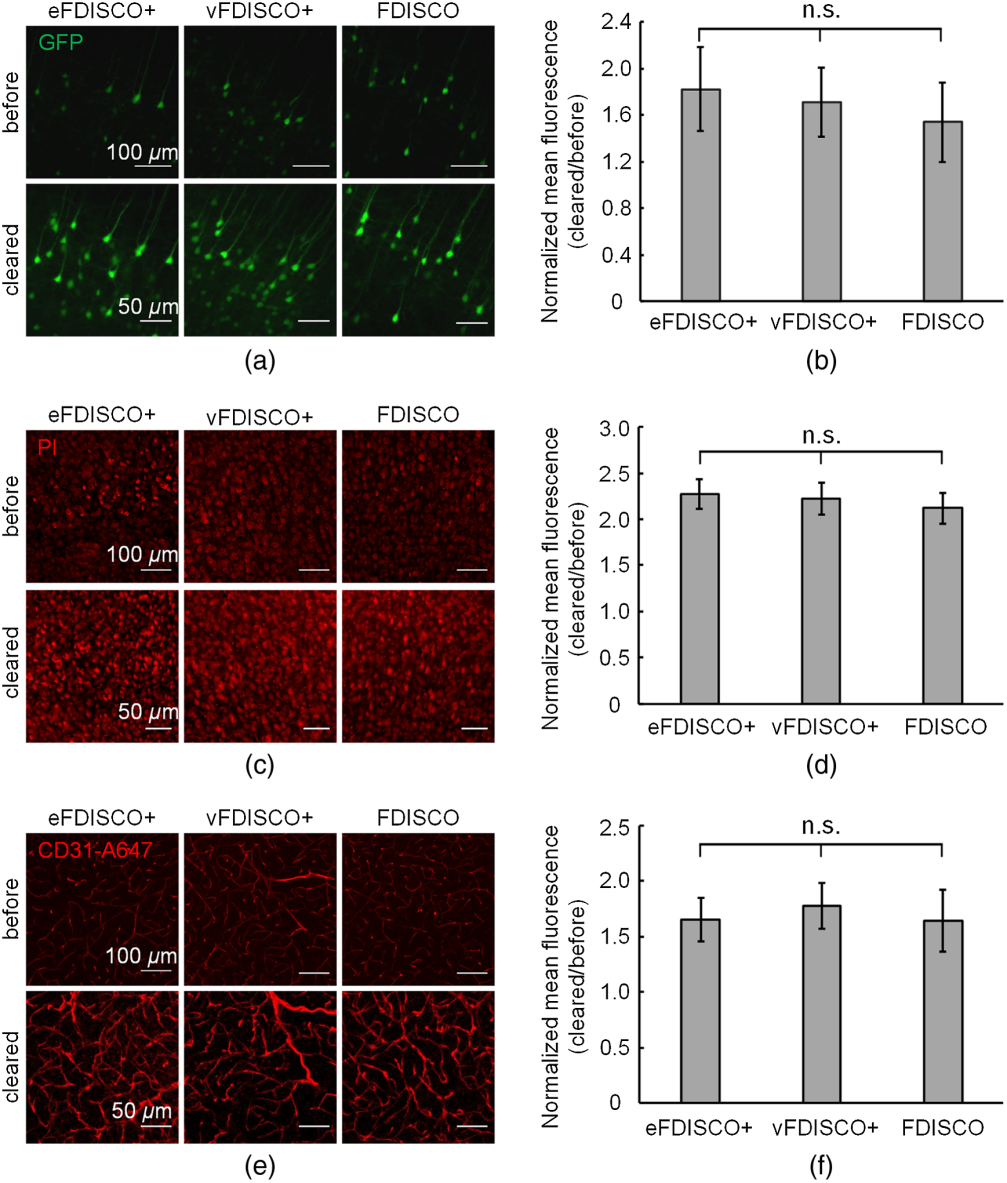
FDISCO+ achieves comparable fluorescence enhancement with FDISCO. (a) Confocal imaging of cortical neurons in adult *Thy1*-GFP-M mouse brains before and after clearing. (b) The normalized mean fluorescence in (a) (n=16). (c) Confocal imaging of PI-stained mouse brain sections before and after clearing. (d) The normalized mean fluorescence in (c) (n=9). (e) Confocal imaging of blood vessels stained with CD31-A647 antibody before and after clearing. (f) The normalized mean fluorescence in (e) (n=9). All values are presented as the mean ± SD. Statistical significance in (b), (d), and (f) (n.s., not significant) was assessed by one-way ANOVA followed by the Bonferroni *post hoc* test.

To further investigate the fluorescence preserving capability of FDISCO+, we cleared the mouse brain samples labeled with different fluorescent probes. We imaged the same cortical areas of 1-mm-thick brain sections before and after clearing by FDISCO+ and FDISCO and quantified the intensity change of fluorescence signals. FDISCO+ showed excellent preservation for all tested fluorescence signals, including signals from endogenous GFP, PI, and CD31-A647, which revealed no obvious difference compared to the original FDISCO. The GFP fluorescence intensity of samples cleared by FDISCO+ increases to 175% to 185% compared to the fluorescence intensity before clearing, as shown in Figs. 2(a) and 2(b). The fluorescence intensity of PI signals after clearing increases to at least 210%, as shown in Figs. 2(c) and 2(d). In addition, FDISCO+ also significantly strengthens the CD31-A647 signals, as shown in Figs. 2(e) and 2(f).

### 3.3 FDISCO+ Allows Long-Time Preservation of GFP Fluorescence at RT

Further, we tested the ability of long-time fluorescence preservation of FDISCO+. We imaged the cortical neurons in *Thy1*-GFP-M mouse brains cleared by FDISCO+ and FDISCO at 0, 3, 7, and 14 days then quantified the fluorescence intensity changes normalized to 0 day.

**Fig. 3:**
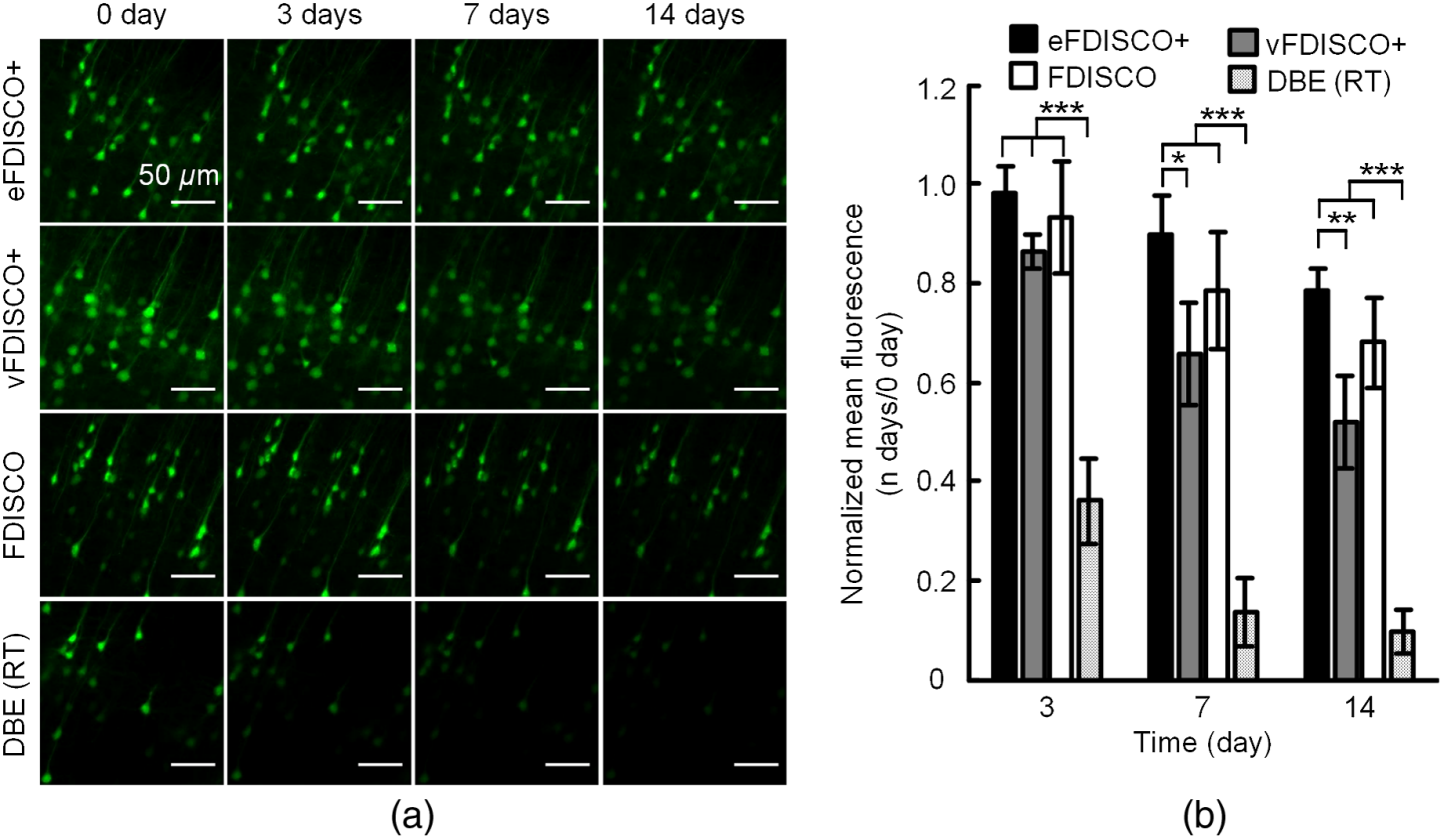
FDISCO+ allows long-time preservation of GFP fluorescence at RT. (a) Confocal imaging of cortical neurons in adult *Thy1*-GFP-M mouse brains cleared after 0, 3, 7, and 14 days using FDISCO+ and FDISCO methods. (b) The histogram of normalized mean fluorescence in (a) (n=6). All values are presented as the mean ± SD. Statistical significance in (b) (*p<0.05; **p<0.01; ***p<0.001) was assessed by one-way ANOVA followed by the Bonferroni *post hoc* test.

Figure 3 shows the representative images and the quantitative data of intensity changes. The GFP fluorescence of brain blocks treated by FDISCO+ and FDISCO are preserved very well for at least two weeks compared to the pure DBE (RT) group. eFDISCO+ shows similar fluorescence preservation with FDISCO and better fluorescence preservation than vFDISCO+ after 7 days with a significant difference, indicating that eFDISCO+ is better in long-time fluorescence preservation than vFDISCO+.

### 3.4 FDISCO+ Shows Good Clearing Capability and Enables Visualization of the Neural Structures

**Fig. 4:**
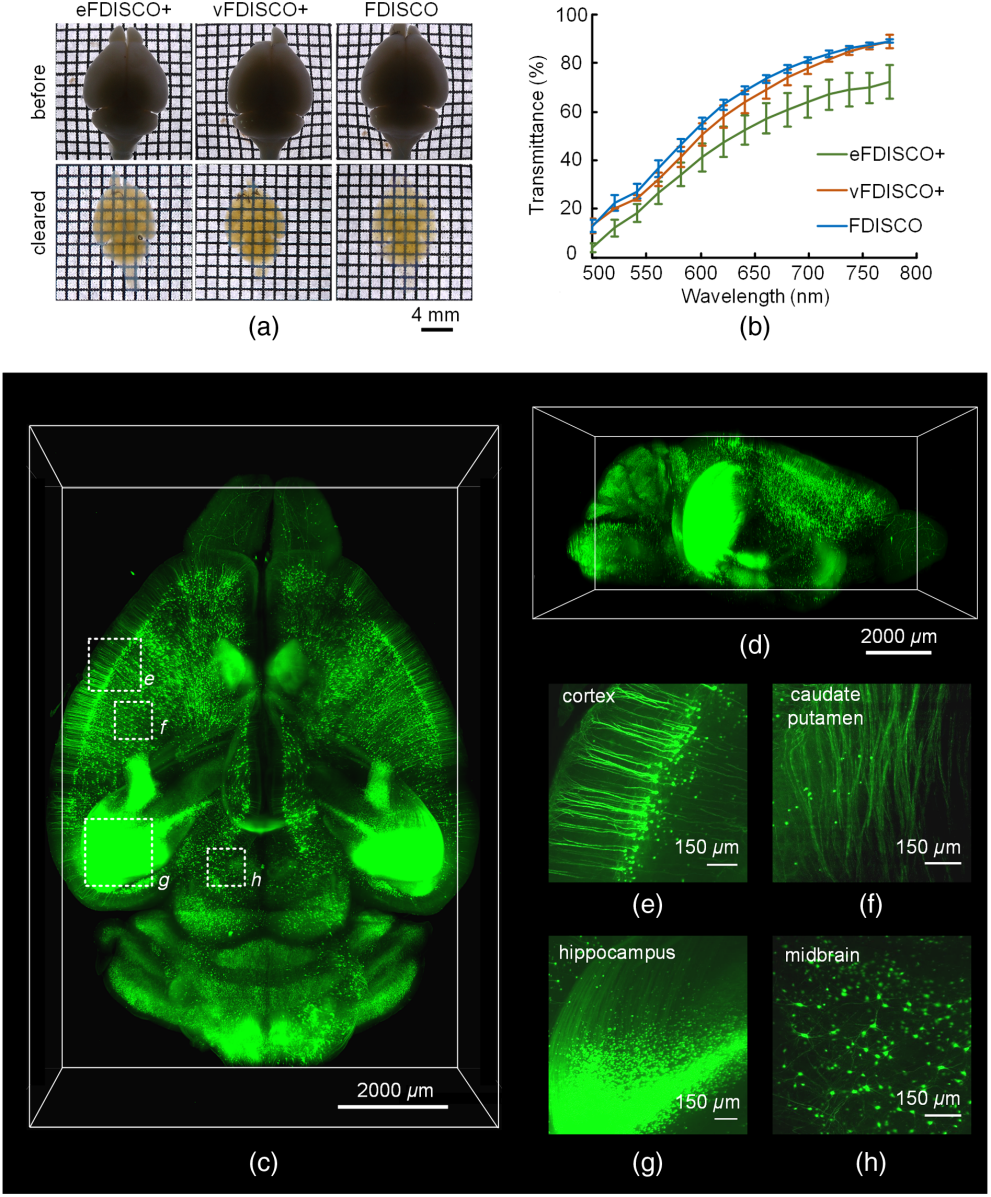
FDISCO+ shows good clearing capability and enables visualization of neural structures. (a) Bright-field images of whole brains before and after clearing. (b) The transmittance curves of cleared mouse brains (C57BL/6J mice, 8 weeks old) (n=3). (c), (d) Images of the whole brain (*Thy1*-GFP-M, 6 weeks old) cleared by vFDISCO+. (e)–(h) The high-magnification images reveal fine structures of different brain areas in (c), including (e) the cortex, (f) caudate putamen, (g) hippocampus, and (h) midbrain. All values are presented as the mean ± SD (Video 1, MP4, 9821 kB [URL: https://doi.org/10.1117/1.NPh.8.3.035007.1]) and (Video 2, MP4, 7244 kB [URL: https://doi.org/10.1117/1.NPh.8.3.035007.2]).

Finally, we tested the clearing capability of FDISCO+ and displayed 3D visualization of the nervous system with LSFM imaging. Figures 4(a) and 4(b) show the bright-field images of whole brains before and after FDISCO+ and FDISCO clearing and the transmittance curves with wavelength. vFDISCO+ achieves similar transparency as FDISCO, and eFDISCO+ displays modest clearing ability for the mouse brain. Meanwhile, we imaged the *Thy1*-GFP-M mouse brain cleared by vFDISCO+ and performed 3D reconstruction from different views, as shown in Figs. 4(c) and 4(d) and Video 1. The fine neuronal structures in different brain regions, such as the cortex, caudate putamen, hippocampus, and midbrain, can be detected effectively, as shown in Figs. 4(e)–4(h). We also obtained the 3D reconstruction of neurons in half brain cleared by eFDISCO+, as shown in Video 2.

## 4 Discussions and Conclusions

In this study, we proposed FDISCO+ for robust fluorescence preservation of cleared samples, addressing the issue that FDISCO-cleared samples could not preserve endogenous fluorescent signals well at RT. FDISCO+ shows comparable fluorescence enhancement to FDISCO and achieves long-time fluorescence preservation, enabling 3D visualization of neuronal structures in the mouse brain.

Generally, the environmental temperature of fluorescence microscopy is at RT, which conflicts with the storage condition of FDISCO. Though a short exposure time under RT is acceptable, a long-time imaging process is inevitable when it comes to large-volume imaging at high resolution. Some precious samples usually need to be imaged many times. In this case, the fluorescence decrease of FDISCO-cleared samples at RT would impede the 3D imaging and visualization of intact organs or tissues. FDISCO+ eliminates this conflict by realizing the robust fluorescence preservation of cleared samples under RT, making it convenient for long-time storage and long-distance transportation.

As previously mentioned, the peroxides continuously produced in DBE could be the culprit of endogenous fluorescence attenuation.^19^ Hence, we chose two antioxidants that could prevent the peroxide generation in DBE for a long time, involving the metal chelator, EDTP, and the free radical scavenger, vitamin E. Although the EDTP has been used to create an alkaline environment in the CUBIC and PEGASOS,^28^^,^^34^^,^^35^ it has not been reported to be used as the antioxidant in the clearing methods. The EDTP was often used as a chelating agent, and the chelator has been demonstrated to have antioxidant properties.^48^ In this study, we consolidated the antioxidant capacity of the EDTP and firstly used it for inhibiting the generation of peroxides in DBE. The vitamin E has been used in the uDISCO method to scavenge the peroxides in BABB-D (mixing benzyl alcohol, benzyl benzoate, and diphenyl ether),^22^ but it is unclear whether vitamin E also can scavenge the peroxides in DBE. This work demonstrated the ability to inhibit peroxides by adding low-concentration vitamin E to DBE. The addition of propyl gallate used in sDISCO method can efficiently preserve EGFP fluorescence signals, but the specimens should also be kept at LT (4°C) during all incubation steps and storage.^26^ It is worth noting that the DBE at LT also did not produce peroxides for a long time, which might be one of the reasons for the long-time preservation of fluorescence in FDISCO.^29^

It has been reported that THF quenches GFP fluorescence by interrupting the hydrogen bonding of protein molecules, and an alkaline pH and LT environment could reduce the sensitivity of GFP to denaturation by THF.^29^ The basic activated aluminum oxide has been used to remove the peroxides in THF before clearing protocols, and the purchased THF has already been added with 250 ppm BHT (butylated hydroxytoluene) to inhibit the generation of peroxides, so the peroxide in THF is no longer a factor affecting the fluorescent proteins. To preserve the best fluorescence signals, the dehydration procedures of FDISCO+ still adopt the LT and alkaline pH conditions.

To meet users’ different needs, we developed two strategies of FDISCO+: eFDISCO+ and vFDISCO+. The eFDISCO+ shows better fluorescence preservation than vFDISCO+, whereas vFDISCO+ shows better transparency than eFDISCO+. This might be because the EDTP contains too many hydroxyl groups, and these polar groups influence the osmosis of DBE molecules in the tissue. Users can choose different clearing protocols according to their experimental purposes: for large samples, the vFDISCO+ is an excellent choice; for small samples, the eFDISCO+ may be selected for better fluorescence preservation.

In conclusion, the FDISCO+ method is a simple and convenient clearing method with robust preservation condition, and it is expected to facilitate long-time or repetitive imaging.

## Code, Data, and Materials Availability

The data used in the manuscript are available upon reasonable request to the corresponding author.

## References

[1] Richardson D. S. Lichtman J. W., “Clarifying tissue clearing,” Cell 162(2), 246–257 (2015). CELLB5 0092-8674 10.1016/j.cell.2015.06.067 26186186 PMC4537058

[r2] Oh S. W. et al., “A mesoscale connectome of the mouse brain,” Nature 508(7495), 207–214 (2014). 10.1038/nature13186 24695228 PMC5102064

[r3] Zingg B. et al., “Neural networks of the mouse neocortex,” Cell 156(5), 1096–1111 (2014). CELLB5 0092-8674 10.1016/j.cell.2014.02.023 24581503 PMC4169118

[r4] Yin X. F. et al., “Spatial distribution of motor endplates and its adaptive change in skeletal muscle,” Theranostics 9(3), 734–746 (2019). 10.7150/thno.28729 30809305 PMC6376466

[5] Zhu J. et al., “MACS: rapid aqueous clearing system for 3D mapping of intact organs,” Adv. Sci. 7(8), 1903185 (2020). 10.1002/advs.201903185 PMC7175264 32328422

[6] Conchello J. A. Lichtman J. W., “Optical sectioning microscopy,” Nat. Methods 2(12), 920–931 (2005).1548-7091 10.1038/nmeth815 16299477

[r7] Mertz J., “Optical sectioning microscopy with planar or structured illumination,” Nat. Methods 8(10), 811–819 (2011).1548-7091 10.1038/nmeth.1709 21959136

[8] Reynaud E. G. et al., “Light sheet-based fluorescence microscopy: more dimensions, more photons, and less photodamage,” HFSP J. 2(5), 266–275 (2008). HJFOA5 1955-2068 10.2976/1.2974980 19404438 PMC2639947

[9] Lichtman J. W. Conchello J. A., “Fluorescence microscopy,” Nat. Methods 2(12), 910–919 (2005).1548-7091 10.1038/nmeth817 16299476

[r10] Zhang B. et al., “Multidither coherent optical adaptive technique for deep tissue two-photon microscopy,” J. Innov. Opt. Health Sci. 12(4), 1942003 (2019). 10.1142/S1793545819420033

[r11] Vannhu L., “Optimization method to suppress background for imaging multiple planes,” J. Innov. Opt. Health Sci. 12(2), 1950004 (2019). 10.1142/S1793545819500044

[12] Nie J. et al., “Fast, 3D isotropic imaging of whole mouse brain using multiangle-resolved subvoxel SPIM,” Adv. Sci. 7(3), 1901891 (2020). 10.1002/advs.201901891 PMC7001627 32042557

[13] Zhu D. et al., “Recent progress in tissue optical clearing,” Laser Photonics Rev. 7(5), 732–757 (2013). 10.1002/lpor.201200056 PMC3856422 24348874

[14] Yu T. et al., “Optical clearing for multiscale biological tissues,” J. Biophotonics 11(2), e201700187 (2018). 10.1002/jbio.201700187 29024450

[15] Costantini I. et al., “In-vivo and ex-vivo optical clearing methods for biological tissues: review,” Biomed. Opt. Express 10(10), 5251–5267 (2019). BOEICL 2156-7085 10.1364/BOE.10.005251 31646045 PMC6788593

[16] Yu T. et al., “Physical and chemical mechanisms of tissue optical clearing,” iScience 24(3), 102178 (2021). 10.1016/j.isci.2021.102178 33718830 PMC7920833

[17] Dodt H.-U. et al., “Ultramicroscopy: three-dimensional visualization of neuronal networks in the whole mouse brain,” Nat. Methods 4(4), 331–336 (2007).1548-7091 10.1038/nmeth1036 17384643

[18] Erturk A. et al., “Three-dimensional imaging of the unsectioned adult spinal cord to assess axon regeneration and glial responses after injury,” Nat. Med. 18(1), 166–171 (2012).1078-8956 10.1038/nm.2600 22198277

[r19] Erturk A. et al., “Three-dimensional imaging of solvent-cleared organs using 3DISCO,” Nat. Protoc. 7(11), 1983–1995 (2012).1754-2189 10.1038/nprot.2012.119 23060243

[20] Becker K. et al., “Chemical clearing and dehydration of GFP expressing mouse brains,” PLoS One 7(3), e33916 (2012). POLNCL 1932-6203 10.1371/journal.pone.0033916 22479475 PMC3316521

[21] Renier N. et al., “iDISCO: a simple, rapid method to immunolabel large tissue samples for volume imaging,” Cell 159(4), 896–910 (2014). CELLB5 0092-8674 10.1016/j.cell.2014.10.010 25417164

[22] Pan C. C. et al., “Shrinkage-mediated imaging of entire organs and organisms using uDISCO,” Nat. Methods 13(10), 859–867 (2016).1548-7091 10.1038/nmeth.3964 27548807

[23] Schwarz M. K. et al., “Fluorescent-protein stabilization and high-resolution imaging of cleared, intact mouse brains,” PLoS One 10(5), e0124650 (2015). POLNCL 1932-6203 10.1371/journal.pone.0124650 25993380 PMC4439039

[24] Klingberg A. et al., “Fully automated evaluation of total glomerular number and capillary tuft size in nephritic kidneys using lightsheet microscopy,” J. Am. Soc. Nephrol. 28(2), 452–459 (2017). JASNEU 1046-6673 10.1681/ASN.2016020232 27487796 PMC5280021

[25] Cai R. Y. et al., “Panoptic imaging of transparent mice reveals whole-body neuronal projections and skull-meninges connections,” Nat. Neurosci. 22(2), 317–327 (2019). NANEFN 1097-6256 10.1038/s41593-018-0301-3 30598527 PMC6494982

[26] Hahn C. et al., “High-resolution imaging of fluorescent whole mouse brains using stabilised organic media (sDISCO),” J. Biophotonics 12(8), e201800368 (2019). 10.1002/jbio.201800368 30932329

[27] Li Y. et al., “Optimization of GFP fluorescence preservation by a modified uDISCO clearing protocol,” Front. Neuroanat. 12, 67 (2018). 10.3389/fnana.2018.00067 30158858 PMC6104128

[28] Jing D. et al., “Tissue clearing of both hard and soft tissue organs with the PEGASOS method,” Cell Res. 28(8), 803–818 (2018).1001-0602 10.1038/s41422-018-0049-z 29844583 PMC6082844

[29] Qi Y. S. et al., “FDISCO: advanced solvent-based clearing method for imaging whole organs,” Sci. Adv. 5(1), eaau8355 (2019). STAMCV 1468-6996 10.1126/sciadv.aau8355 30746463 PMC6357753

[30] Hama H. et al., “Sca*l*eS: an optical clearing palette for biological imaging,” Nat. Neurosci. 18(10), 1518–1529 (2015). NANEFN 1097-6256 10.1038/nn.4107 26368944

[31] Ke M. T. Fujimoto S. Imai T., “SeeDB: a simple and morphology-preserving optical clearing agent for neuronal circuit reconstruction,” Nat. Neurosci. 16(8), 1154–1161 (2013). NANEFN 1097-6256 10.1038/nn.3447 23792946

[32] Ke M. T. et al., “Super-resolution mapping of neuronal circuitry with an index-optimized clearing agent,” Cell Rep. 14(11), 2718–2732 (2016). 10.1016/j.celrep.2016.02.057 26972009

[33] Kuwajima T. et al., “ClearT: a detergent- and solvent-free clearing method for neuronal and non-neuronal tissue,” Development 140(6), 1364–1368 (2013). 10.1242/dev.091844 23444362 PMC3912244

[34] Susaki E. A. et al., “Whole-brain imaging with single-cell resolution using chemical cocktails and computational analysis,” Cell 157(3), 726–739 (2014). CELLB5 0092-8674 10.1016/j.cell.2014.03.042 24746791

[r35] Tainaka K. et al., “Whole-body imaging with single-cell resolution by tissue decolorization,” Cell 159(4), 911–924 (2014). CELLB5 0092-8674 10.1016/j.cell.2014.10.034 25417165

[36] Susaki E. A. et al., “Advanced CUBIC protocols for whole-brain and whole-body clearing and imaging,” Nat. Protoc. 10(11), 1709–1727 (2015).1754-2189 10.1038/nprot.2015.085 26448360

[37] Li W. Germain R. N. Gerner M. Y., “Multiplex, quantitative cellular analysis in large tissue volumes with clearing-enhanced 3D microscopy (Ce3D),” Proc. Natl. Acad. Sci. U. S. A. 114(35), E7321–E7330 (2017). 10.1073/pnas.1708981114 28808033 PMC5584454

[38] Yu T. et al., “RTF: a rapid and versatile tissue optical clearing method,” Sci. Rep. 8, 1964 (2018). SRCEC3 2045-2322 10.1038/s41598-018-20306-3 29386656 PMC5792593

[39] Chung K. et al., “Structural and molecular interrogation of intact biological systems,” Nature 497(7449), 332 (2013). 10.1038/nature12107 23575631 PMC4092167

[40] Tomer R. et al., “Advanced CLARITY for rapid and high-resolution imaging of intact tissues,” Nat. Protoc. 9(7), 1682–1697 (2014).1754-2189 10.1038/nprot.2014.123 24945384 PMC4096681

[41] Yang B. et al., “Single-cell phenotyping within transparent intact tissue through whole-body clearing,” Cell 158(4), 945–958 (2014). CELLB5 0092-8674 10.1016/j.cell.2014.07.017 25088144 PMC4153367

[r42] Treweek J. B. et al., “Whole-body tissue stabilization and selective extractions via tissue-hydrogel hybrids for high-resolution intact circuit mapping and phenotyping,” Nat. Protoc. 10(11), 1860–1896 (2015).1754-2189 10.1038/nprot.2015.122 26492141 PMC4917295

[43] Yu T. et al., “Elevated-temperature-induced acceleration of PACT clearing process of mouse brain tissue,” Sci. Rep. 7, 38848 (2017). SRCEC3 2045-2322 10.1038/srep38848 28139694 PMC5282525

[44] Wan P. et al., “Evaluation of seven optical clearing methods in mouse brain,” Neurophotonics 5(3), 035007 (2018). 10.1117/1.NPh.5.3.035007 30155510 PMC6109056

[45] Fang C. et al., “Minutes-timescale 3D isotropic imaging of entire organs at subcellular resolution by content-aware compressed-sensing light-sheet microscopy,” Nat. Commun. 12(1), 107 (2021). NCAOBW 2041-1723 10.1038/s41467-020-20329-3 33398061 PMC7782498

[46] Yu T. et al., “Rapid and prodium iodide-compatible optical clearing method for brain tissue based on sugar/sugar-alcohol,” J. Biomed. Opt. 21(8), 081203 (2016). JBOPFO 1083-3668 10.1117/1.JBO.21.8.081203 26968577

[47] Otsu N., “A threshold selection method from gray-level histograms,” IEEE Trans. Syst. Man Cybern. Syst. 9, 62–66 (1979). 10.1109/TSMC.1979.4310076

[48] Balcerczyk A. Sowa K. Bartou G., “Metal chelators react also with reactive oxygen and nitrogen species,” Biochem. Biophys. Res. Commun. 352(2), 522–525 (2007). BBRCA9 0006-291X 10.1016/j.bbrc.2006.11.053 17126814

